# Associations Between Systemic Inflammatory Markers, Metabolic Dysfunction, and Liver Fibrosis Scores in Patients with MASLD

**DOI:** 10.3390/metabo16010025

**Published:** 2025-12-25

**Authors:** Ragaey Ahmad Eid, Ahmed Moheyeldien Hamed, Sara O. Elgendy, Khalid M. Orayj, Ahmed R. N. Ibrahim, Ahmed M. Abdel Hamied, Engy A. Wahsh, Maha Youssif, Hoda Rabea, Yasmin M. Madney, Dina Attia, Shaymaa Nafady

**Affiliations:** 1 Department of Gastroenterology, Hepatology, and Infectious Diseases (Tropical Medicine Department), Faculty of Medicine, Beni-Suef University, Beni-Suef 62511, Egypt; dina.attia@med.bsu.edu.eg (D.A.); shaymaanafady@med.bsu.edu.eg (S.N.); 2 Department of Internal Medicine, Faculty of Medicine, Beni-Suef University, Beni-Suef 62511, Egypt; moheyinternist@med.bsu.edu.eg; 3 Clinical and Chemical Pathology Department, Faculty of Medicine, Beni-Suef University, Beni-Suef 62511, Egypt; 4 Department of Clinical Pharmacy, College of Pharmacy, King Khalid University, Abha 62521, Saudi Arabia; korayg@kku.edu.sa (K.M.O.); aribrahim@kku.edu.sa (A.R.N.I.); 5 Department of Pediatrics, Faculty of Medicine, Nahda University (NUB), Beni-Suef 19206, Egypt; ahmed.abdelhamid@nub.edu.eg; 6 Department of Clinical Pharmacy, Faculty of Pharmacy, October 6 University, Giza 12585, Egypt; engywahsh@o6u.edu.eg (E.A.W.); maha.youssif.ph@o6u.edu.eg (M.Y.); 7 Department of Clinical pharmacy, Faculty of Pharmacy, Beni-Suef University, Beni-Suef 62511, Egypt; hoda.ahmed@pharm.bsu.edu.eg (H.R.); yasminmohamed5050@pharm.bsu.edu.eg (Y.M.M.)

**Keywords:** metabolic dysfunction-associated steatotic liver disease (MASLD), IL-6, hsCRP, TNF-α, inflammatory markers

## Abstract

Background: Metabolic dysfunction-associated steatotic liver disease (MASLD) has emerged as a global health challenge due to its complex pathophysiological processes. Systemic inflammation may profoundly affect disease progression, but the correlation between inflammatory markers and disease severity remains inadequately explored. This cross-sectional analysis within a prospective cohort evaluated associations of inflammatory markers (IL-6, TNF-α, hsCRP) with MASLD severity (five non-invasive scores) and metabolic indices, primarily with early-stage disease (66.7% mild fibrosis by TE). Methods: We recruited 120 patients diagnosed with MASLD. Assessment included anthropometric measurements, laboratory analyses, and non-invasive fibrosis evaluation using five validated scoring systems (APRI, FIB-4, NAFLD fibrosis score, FAST score, and transient elastography). Inflammatory markers were quantified using high-sensitivity ELISA techniques. Medication/comorbidities were recorded (statins 23.3%, diabetes drugs 26.7%), and multivariate regressions and FDR correction were applied. Results: Patients showed remarkably elevated inflammatory markers compared to reference ranges: IL-6 (15.1 ± 9.3 pg/mL), TNF-α (38.8 ± 29.1 pg/mL), and hsCRP (12.3 ± 11.1 mg/L). No correlations were found between inflammatory markers and disease severity across any non-invasive scoring system. However, TNF-α correlated significantly with waist circumference (r = 0.28, *p* = 0.002) and ALT (r = 0.19, *p* = 0.03), while showing inverse correlations with total cholesterol (r = −0.27, *p* = 0.03) and LDL (r = −0.22, *p* = 0.02). In contrast, hsCRP correlated positively with LDL (r = 0.20, *p* = 0.02) and WBC count (r = 0.24, *p* = 0.008). Conclusion: This study reveals a dissociation between systemic inflammatory markers and hepatic fibrosis severity in MASLD. Inflammatory Markers showed stronger metabolic associations than fibrosis, limiting their utility as fibrosis surrogates in early MASLD. These findings support a dual-pathway approach to MASLD management, targeting metabolic and hepatic components independently. The divergent associations of TNF-α and hsCRP with lipid profiles suggest distinct inflammatory mechanisms in MASLD.

## 1. Introduction

Metabolic dysfunction-associated steatotic liver disease (MASLD) has emerged as the most prevalent chronic liver disease globally, affecting approximately 38% of adults worldwide, with projections suggesting this prevalence will exceed 55% by 2040 [1].

Beyond hepatic involvement, MASLD is correlated with a multisystem metabolic disorder intricately linked to insulin resistance, obesity, dyslipidemia, hypertension, and genetic susceptibility [2]. These factors contribute to disease progression from simple steatosis to metabolic dysfunction-associated steatohepatitis (MASH), advanced fibrosis, cirrhosis, and hepatocellular carcinoma [3], while also exacerbating systemic complications including cardiovascular disease, atherosclerosis, chronic kidney disease, and metabolic syndrome [4].

A central feature linking MASLD to its systemic manifestations is chronic low-grade systemic inflammation. Adipose tissue dysfunction, insulin resistance, and metabolic dysregulation trigger activation of innate immune pathways and release of pro-inflammatory cytokines into circulation [5]. Among these key inflammatory mediators, interleukin-6 (IL-6), tumour necrosis factor-alpha (TNF-α), and high-sensitivity C-reactive protein (hsCRP) are well-established biomarkers of systemic inflammation implicated in both metabolic disorders and cardiovascular disease, as well as in the progression of chronic liver disease [6,7]. However, the intricate relationships between systemic inflammatory markers, hepatic disease severity, and metabolic dysfunction remain incompletely understood, particularly regarding whether these biomarkers showed stronger associations with liver pathology or extra-hepatic metabolic disturbances [8].

While liver biopsy remains the gold standard for assessing MASLD severity, its invasiveness, sampling variability, cost, and risk of complications limit its routine clinical application [9]. This has driven the development and validation of numerous non-invasive assessment tools, including serum biomarker scores and imaging-based techniques. Among serum-based scores, the Fibrosis-4 Index (FIB-4) and the AST-to-Platelet Ratio Index (APRI) have demonstrated satisfactory diagnostic performance for advanced fibrosis [10,11]. The NAFLD Fibrosis Score (NFS) has shown moderate accuracy for predicting liver-related events [12]. More recently, the FibroScan-AST (FAST) score, combining transient elastography with AST levels, has emerged as a promising tool for non-invasive identification of patients with fibrotic MASH [13].

Despite growing evidence, fundamental uncertainty persists regarding whether inflammatory biomarkers in MASLD showed stronger associations with hepatic disease severity or instead mirror the underlying metabolic dysfunction that accompanies the condition. Most previous studies have examined these markers either in relation to histological severity or cardiovascular outcomes, but few have systematically compared their associations with both comprehensive non-invasive fibrosis indices and metabolic parameters within the same MASLD cohort [14,15]. This distinction has critical implications for patient risk stratification, treatment target selection, and interpretation of therapeutic trials.

The widespread clinical measurement of inflammatory biomarkers in MASLD patients raises a fundamental question: do these biomarkers provide meaningful information about liver disease severity that could guide clinical decisions, or do they show stronger associations with metabolic disturbances that should be addressed through metabolic interventions rather than liver-specific therapies? While cytokines and acute-phase reactants are known to be elevated in MASLD, their precise relationship to fibrosis progression as assessed by the comprehensive array of validated non-invasive scoring systems now available remains inadequately characterized.

The present study addresses this critical knowledge gap by evaluating serum levels of three key inflammatory markers (IL-6, TNF-α, and hsCRP) in a well-characterized cohort of patients with MASLD and systematically analyzing their correlations with: (1) disease severity assessed through five validated non-invasive indices (APRI, FIB-4, NAFLD fibrosis score, FAST score, and transient elastography with controlled attenuation parameter), and (2) key metabolic parameters, including anthropometric indices, glycemic control, and comprehensive lipid profiling. By clarifying these relationships, we aim to determine whether inflammatory markers function as prognostic tools associating with hepatic disease severity or primarily indicate extra-hepatic metabolic dysfunction in MASLD, thereby informing more rational approaches to risk stratification and therapeutic targeting in this increasingly prevalent and clinically heterogeneous condition.

## 2. Materials and Methods

### 2.1. Research Framework and Ethics Protocol

This was a cross-sectional analysis conducted within a prospective observational cohort enrolled between January 2025 and October 2025 at Beni-Suef University Hospital. The study was approved by the institutional ethics committee (approval number: FMB-SUREC/05012025/Eid), and all procedures adhered to Declaration of Helsinki principles. All participants provided written informed consent.

### 2.2. Participant Eligibility Criteria

Participants were required to meet the MASLD diagnostic criteria [16], including confirmation of hepatic steatosis through imaging (abdominal ultrasound) and controlled attenuation parameter (CAP) measurement. Additionally, subjects required at least one metabolic dysfunction indicator: BMI ≥ 25 kg/m^2^, diabetes or impaired glycemic control (defined as HbA1c ≥ 6.5% or fasting plasma glucose ≥ 126 mg/dL), hypertension (≥130/85 mmHg or treated), dyslipidemia (triglycerides ≥ 150 mg/dL or HDL-cholesterol < 40 mg/dL in men/<50 mg/dL in women).

Individuals with alternative steatosis etiologies were excluded, including excessive alcohol consumption (≥50 g/day for women, ≥60 g/day for men), medication-induced hepatic injury, hepatitis B or C infection, or Wilson’s disease. Additional exclusions included age < 18 years, pregnancy, decompensated cirrhosis, portal hypertension, hepatocellular malignancy, and anti-inflammatory medications.

### 2.3. Clinical and Anthropometric Evaluation

All patients underwent comprehensive medical history assessment and physical examination. Anthropometric measurements included body mass index (BMI; weight in kg/height in m^2^) and waist circumference (measured at the midpoint between the lowest rib and iliac crest after normal expiration in a standing position).

#### Medication and Comorbidity Controls

Data on medication use and comorbidities were recorded: statin use (n = 28, 23.3%), diabetes medications (n = 32, 26.7%), antihypertensives (n = 45, 37.5%), and smoking status (n = 18 current smokers, 15.0%). These factors can influence inflammatory markers but were not adjusted in primary univariate analyses due to collinearity with metabolic traits; multivariate models included diabetes status and relevant covariates. Lack of complete adjustment acknowledged as limitation.

### 2.4. Laboratory Investigations

#### 2.4.1. Blood Sample Collection and Processing

Venous blood samples were collected after an overnight fast (minimum 8 h) between 8:00 and 10:00 AM to minimize circadian variation. Blood was drawn using standard venipuncture technique into appropriate collection tubes: serum separator tubes for biochemical analyses, EDTA tubes for complete blood count, and plain tubes for inflammatory marker assessment. Serum was separated by centrifugation at 3000 rpm for 15 min at room temperature within 30 min of collection. Samples were aliquoted and stored at −80 °C until batch analysis to avoid freeze–thaw cycles.

#### 2.4.2. Routine Biochemical Parameters

Liver enzymes (ALT, AST in IU/L), GGT (IU/L), albumin (g/dL), fasting plasma glucose (mg/dL), HbA1c (%), lipid profile (total cholesterol, HDL/LDL-cholesterol, triglycerides in mg/dL), complete blood count (platelets/WBC in 10^9^/L), and renal function were measured using automated analyzers according to standard laboratory protocols. Fasting insulin was measured using chemiluminescence immunoassay, and insulin resistance was calculated using the homeostatic model assessment for insulin resistance (HOMA-IR) formula: [fasting insulin (μU/mL) × fasting glucose (mg/dL)]/405 [17].

#### 2.4.3. Inflammatory Marker Quantification

Interleukin-6 (IL-6): Serum IL-6 levels were quantified using the Quantikine HS High Sensitivity ELISA Kit (R&D Systems, Catalog #HS600C, Minneapolis, MN, USA). The assay procedure followed the manufacturer’s protocol with a total assay time of approximately 4 h. The assay sensitivity was 0.09 pg/mL. Intra-assay and inter-assay coefficients of variation were <7.8% and <7.2%, respectively.

Tumor Necrosis Factor-alpha (TNF-α): Serum TNF-α concentrations were determined using the Quantikine HS ELISA Kit (R&D Systems, Catalog #HSTA00E, Minneapolis, MN, USA) following the manufacturer’s instructions. The assay sensitivity was 0.049 pg/mL, with intra-assay and inter-assay precision (CV%) of <8.7% and <10.8%, respectively. The detection range was 0.5–32 pg/mL.

High-Sensitivity C-Reactive Protein (hsCRP): HsCRP was measured using a high-sensitivity turbidimetric immunoassay (Siemens Healthcare Diagnostics, Tarrytown, NY, USA). The assay range was 0.1–20 mg/L with functional sensitivity of <0.3 mg/L. Intra-assay and inter-assay imprecision were <5% across the measuring range.

All inflammatory marker measurements were performed in duplicate, and mean values were used for statistical analysis. Samples with coefficient of variation >10% between duplicates were re-analyzed. Quality control procedures included analysis of low, medium, and high control samples with each batch.

### 2.5. Hepatic Steatosis and Fibrosis Assessment

#### 2.5.1. Abdominal Ultrasonography

Abdominal ultrasonography was conducted using a Philips EPIQ 7G system (Philips, Bothell, WA, U.S.A) following a standardized protocol administered by two experienced radiologists. The examination evaluated liver echogenicity, hepatomegaly, signs of portal hypertension, and focal hepatic lesions. Ultrasonographic grading of hepatic steatosis was determined according to the following criteria: Grade 0 (Normal) denoted normal liver echogenicity; Grade 1 (Mild) was characterized by minimal diffuse increase in hepatic echogenicity with preserved visualization of the diaphragm and intrahepatic vessel borders; Grade 2 (Moderate) was defined by moderate increase in hepatic echogenicity with slightly impaired visualization of intrahepatic vessels and diaphragm; Grade 3 (Severe) was identified by marked increase in echogenicity with poor or absent visualization of hepatic vessels and diaphragm [4].

#### 2.5.2. Transient Elastography (FibroScan^®^)

Liver stiffness measurement (LSM) and controlled attenuation parameter (CAP) measurement were performed using a FibroScan^®^ 502 Touch device (Echosens, Paris, France) equipped with M and XL probes. Examinations were conducted by trained operators, with patients fasting for at least 3 h prior to examination. Patients were positioned supine with the right arm in maximal abduction, and measurements were obtained through intercostal spaces targeting the right hepatic lobe. CAP, representing ultrasound attenuation at 3.5 MHz and expressed in dB/m, provided steatosis assessment. LSM, expressed in kilopascals (kPa), evaluated liver stiffness as a surrogate for fibrosis. Only examinations with at least 10 valid measurements, success rate ≥ 60%, and interquartile range (IQR)/median ratio < 30% were considered reliable and included in analysis [18].

### 2.6. Non-Invasive Fibrosis Score Calculations: Multiple Validated Non-Invasive Scoring Systems Were Calculated for Each Patient

APRI (AST-to-Platelet Ratio Index): APRI = [(AST [IU/L]/AST upper normal limit [40 IU/L]) × 100]/Platelet count [10^9^/L]. Cutoffs: <0.5 excludes significant fibrosis; >1.0 suggests cirrhosis [19].

FIB-4 Index: FIB-4 = [Age (years) × AST (IU/L)]/[Platelet count (10^9^/L) × √ALT (IU/L)]. Cutoffs: <1.45 excludes advanced fibrosis (NPV 90%); >3.25 indicates advanced fibrosis with 97% specificity [20].

NAFLD Fibrosis Score (NFS): NFS = −1.675 + 0.037 × Age (years) + 0.094 × BMI (kg/m^2^) + 1.13 × IFG/Diabetes (yes = 1, no = 0) + 0.99 × AST/ALT ratio − 0.013 × Platelet count (10^9^/L) − 0.66 × Albumin (g/dL). Cutoffs: <−1.455 excludes advanced fibrosis; >0.676 indicates advanced fibrosis [21].

FAST Score (FibroScan-AST): The FAST score was calculated using the proprietary formula combining LSM (kPa), CAP (dB/m), and AST (IU/L) as provided by the FibroScan^®^ device software (Version C 3.1., Echosens, Paris, France) Cutoffs: <0.35 rules out fibrotic NASH; >0.67 rules in fibrotic NASH [22].

ASCVD Risk Score: The 10-year atherosclerotic cardiovascular disease risk was calculated using the Pooled Cohort Equations from the 2013 ACC/AHA guidelines, incorporating age, sex, race, total cholesterol, HDL-cholesterol, systolic blood pressure, antihypertensive medication use, diabetes status, and smoking status. The score estimates the 10-year risk of first hard ASCVD event (nonfatal myocardial infarction, CHD death, or fatal/nonfatal stroke) [23].

### 2.7. Statistical Analysis

Data analysis was performed using Statistical Package for Social Sciences (SPSS) software version 22 (SPSS Inc., Chicago, IL, USA). Descriptive analysis included frequencies and percentages for qualitative data. Continuous variables were tested for normality using the Kolmogorov–Smirnov test. Parametric data were expressed as means with standard deviations, while non-parametric data were reported as medians with ranges.

The Kruskal–Wallis test was used to compare more than two independent groups for non-parametric data. The Mann–Whitney U test compared two independent groups. Chi-square test (or Fisher’s exact test when appropriate) compared categorical variables. Spearman’s rank correlation coefficient assessed associations between non-parametric variables and between inflammatory markers with clinical, laboratory, and fibrosis score parameters.

All statistical tests were two-sided, and a *p*-value < 0.05 was considered statistically significant. Multivariate Analysis: Linear regressions with inflammatory markers as dependents, adjusted for age, sex, BMI, waist circumference, diabetes status, ALT/AST. Benjamini–Hochberg FDR correction applied to correlations (q < 0.05 significant).

## 3. Results

Our analysis proceeded in three sequential phases: first, we characterized the demographic and clinical features of our MASLD cohort; second, we systematically evaluated disease severity using multiple validated non-invasive scoring systems; and third, we examined relationships between inflammatory markers and both fibrosis severity and metabolic parameters:

### 3.1. Demographic and Clinical Characteristics

Our study included 120 patients with MASLD, with a mean age of 47.3 ± 7.9 years (range: 29–66 years). The cohort demonstrated female predominance (56.7%, n = 68), and 30% (n = 36) had diabetes mellitus. Anthropometric measurements revealed substantial metabolic burden, with mean waist circumference of 111.4 ± 11.9 cm (range: 84–176 cm) and mean BMI of 35.2 ± 7.3 kg/m^2^ (range: 24.4–61.8 kg/m^2^), indicating obesity as a predominant feature in this population [Table 1]

The laboratory parameters associated with the metabolic dysfunction characteristic of MASLD were as follows: Mean fasting blood glucose was 120.02 ± 47.9 mg/dL (range: 60–372 mg/dL), with mean HOMA-IR of 2.2 ± 0.98 (range: 0.86–6.4), indicating significant insulin resistance. Liver enzymes showed mild elevation, with mean ALT of 33.1 ± 14.1 U/L (range: 16–85 U/L) and mean AST of 33.5 ± 11.2 U/L (range: 15–75 U/L). Lipid profile abnormalities were common, with mean total cholesterol of 191.1 ± 46.3 mg/dL, triglycerides of 196.3 ± 101.2 mg/dL, LDL-cholesterol of 103.6 ± 47.7 mg/dL, and HDL-cholesterol of 47.6 ± 9.9 mg/dL. Hematological parameters included mean hemoglobin of 13.3 ± 1.5 g/dL, platelet count of 271.4 ± 59.5 × 10^9^/L, and white blood cell count of 6.9 ± 2.3 × 10^9^/L. Complete demographic and laboratory data are presented in [Table 1].

### 3.2. Assessment of MASLD Severity Using Non-Invasive Parameters

Hepatic steatosis and fibrosis were assessed using multiple validated non-invasive tools. The median CAP score was 334 dB/m (range: 100–400 dB/m), with the majority of patients (83.3%, n = 100) classified as having severe steatosis (S3), while 6.7% (n = 8) had S0, 3.3% (n = 4) had S1, and 6.7% (n = 8) had S2 steatosis [Table 2].

Regarding fibrosis assessment, transient elastography revealed a median liver stiffness measurement of 6.1 kPa (range: 2.1–28 kPa). The distribution of fibrosis severity by TE score showed that 66.7% (n = 80) had mild fibrosis, 23.3% (n = 28) had moderate fibrosis, and 10% (n = 12) had severe fibrosis. The FAST score (median: 0.33, range: 0.04–18) classified 99.2% (n = 119) as mild and only 0.8% (n = 1) as severe [Table 2].

Serum-based fibrosis scores demonstrated varying severity distributions. The APRI score (median: 0.30, range: 0.1–1.1) categorized 95% (n = 114) as low risk and 5% (n = 6) as intermediate risk. The FIB-4 score (median: 1.01, range: 0.43–3.96) identified 71.7% (n = 86) as having mild fibrosis, 25.8% (n = 31) as moderate, and 2.5% (n = 3) as severe. The NAFLD fibrosis score (median: −1.17, range: −3.86 to 2.15) classified 38.3% (n = 46) as low risk, 32.5% (n = 39) as intermediate risk, and 29.2% (n = 35) as high risk. The ASCVD score (median: 2, range: 0.3–35.3) categorized 80% (n = 96) as low risk, 9.2% (n = 11) as borderline, 8.3% (n = 10) as intermediate risk, and 2.5% (n = 3) as high risk. Complete severity assessment data are shown in [Table 2].

### 3.3. Inflammatory Marker Profiles in MASLD Patients

**Figure 1:**
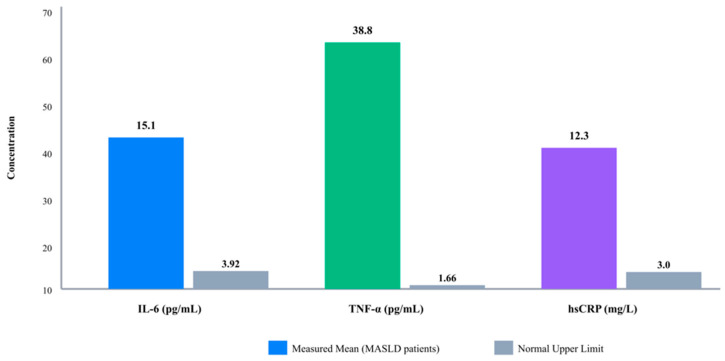
Inflammatory Marker Profiles in MASLD Patients.

Analysis of systemic inflammatory markers revealed markedly elevated levels compared to established reference ranges. Mean IL-6 level was 15.1 ± 9.3 pg/mL (median: 14.4 pg/mL, range: 0–68.3 pg/mL), substantially exceeding the normal reference range of 0.495–3.92 pg/mL. Mean TNF-α concentration was 38.8 ± 29.1 pg/mL (median: 30.4 pg/mL, range: 0–109.6 pg/mL), markedly higher than the normal range of 0.753–1.66 pg/mL. Mean hsCRP level was 12.3 ± 11.1 mg/L (median: 8.4 mg/L, range: 0.5–44.9 mg/L), well above the elevated cardiovascular risk threshold of >3.0 mg/L [Figure 1]. Levels relative to references, not matched controls—interpret magnitude cautiously. These findings confirm substantial systemic inflammation in our MASLD cohort.

### 3.4. Relationship Between IL-6 and Hepatic Fibrosis Severity

To determine whether IL-6 levels correlate with liver disease severity, we systematically analyzed IL-6 concentrations across all seven non-invasive fibrosis scoring systems. Contrary to expectations, IL-6 demonstrated no significant associations with fibrosis severity across any of the assessment tools [Table S1].

When stratified by CAP score for steatosis severity, IL-6 levels showed no significant differences between S0 (13.6 ± 3.2 pg/mL), S1 (16.3 ± 11.3 pg/mL), S2 (12.5 ± 7.1 pg/mL), and S3 (15.4 ± 9.8 pg/mL) groups (*p* = 0.86). Similarly, APRI score categories revealed comparable IL-6 levels between low-risk (14.9 ± 9.5 pg/mL) and intermediate-risk (17.1 ± 3.5 pg/mL) groups (*p* = 0.22). The NAFLD fibrosis score categories demonstrated no significant variation in IL-6 levels across low-risk (15.9 ± 11.7 pg/mL), intermediate-risk (13.6 ± 6.9 pg/mL), and high-risk (15.5 ± 8.2 pg/mL) groups (*p* = 0.70) [Table S1].

Transient elastography-based classification showed no significant IL-6 differences between mild (14.8 ± 10.3 pg/mL), moderate (14.7 ± 7.4 pg/mL), and severe (17.8 ± 6.4 pg/mL) fibrosis (*p* = 0.75). The FAST score comparison between mild (15.1 ± 9.4 pg/mL) and severe (12.7 ± 0.0 pg/mL) categories was also non-significant (*p* = 0.73). FIB-4 score categories showed no significant differences across mild (14.8 ± 9.8 pg/mL), moderate (15.4 ± 8.2 pg/mL), and severe (19.3 ± 4.4 pg/mL) fibrosis (*p* = 0.19). Finally, ASCVD risk categories demonstrated no significant variation in IL-6 levels across low (14.3 ± 8.7 pg/mL), borderline (15.8 ± 14.4 pg/mL), intermediate (18.7 ± 7.2 pg/mL), and high (22.9 ± 9.8 pg/mL) risk groups (*p* = 0.15) [Table S1].

The fibrosis scores entered in the multivariate models all had β < 0.10 and *p* > 0.20 (nonsignificant).

### 3.5. Relationship Between hsCRP and Hepatic Fibrosis Severity

High-sensitivity C-reactive protein levels were similarly analyzed across all fibrosis scoring systems, revealing consistent lack of association with disease severity [Table S2].

CAP score stratification showed no significant hsCRP differences between S0 (19.1 ± 19.9 mg/L), S1 (6.7 ± 3.8 mg/L), S2 (15.2 ± 13.4 mg/L), and S3 (11.8 ± 10.0 mg/L) groups (*p* = 0.69). APRI score categories demonstrated comparable hsCRP levels between low-risk (12.4 ± 11.3 mg/L) and intermediate-risk (11.8 ± 4.7 mg/L) groups (*p* = 0.42). The NAFLD fibrosis score revealed no significant differences across low-risk (15.7 ± 13.9 mg/L), intermediate-risk (9.6 ± 7.9 mg/L), and high-risk (10.9 ± 8.5 mg/L) categories (*p* = 0.16) [Table S2].

Transient elastography classification showed no significant hsCRP variation between mild (12.6 ± 11.3 mg/L), moderate (12.1 ± 10.2 mg/L), and severe (10.9 ± 12.1 mg/L) fibrosis groups (*p* = 0.73). FAST score comparison between mild (12.4 ± 11.1 mg/L) and severe (3.9 ± 0.0 mg/L) categories was non-significant (*p* = 0.48). FIB-4 categories demonstrated no significant differences across mild (13.1 ± 11.7 mg/L), moderate (10.1 ± 9.4 mg/L), and severe (12.0 ± 5.9 mg/L) fibrosis (*p* = 0.38). ASCVD risk categories also showed no significant hsCRP variation across low (12.8 ± 11.7 mg/L), borderline (13.5 ± 10.3 mg/L), intermediate (8.3 ± 4.4 mg/L), and high (7.8 ± 6.9 mg/L) risk groups (*p* = 0.77) [Table S2].

### 3.6. Relationship Between TNF-α and Hepatic Fibrosis Severity

Tumor necrosis factor-alpha levels were comprehensively evaluated across all non-invasive scoring systems, consistently demonstrating no significant associations with fibrosis severity [Table S3].

When stratified by CAP score, TNF-α levels showed no significant differences between S0 (42.02 ± 35.1 pg/mL), S1 (34.1 ± 20.3 pg/mL), S2 (32.2 ± 24.7 pg/mL), and S3 (39.2 ± 29.5 pg/mL) groups (*p* = 0.91). APRI score categories revealed comparable TNF-α levels between low-risk (38.8 ± 29.3 pg/mL) and intermediate-risk (36.8 ± 27.8 pg/mL) groups (*p* = 0.91). The NAFLD fibrosis score demonstrated no significant variation across low-risk (42.1 ± 31.01 pg/mL), intermediate-risk (33.6 ± 27.4 pg/mL), and high-risk (39.9 ± 28.3 pg/mL) categories (*p* = 0.45) [Table S3].

Transient elastography-based classification showed no significant TNF-α differences between mild (36.1 ± 30.2 pg/mL), moderate (43.1 ± 26.9 pg/mL), and severe (45.8 ± 26.4 pg/mL) fibrosis groups (*p* = 0.17). FAST score comparison between mild (38.3 ± 28.9 pg/mL) and severe (86.6 ± 0.0 pg/mL) categories was non-significant (*p* = 0.17). FIB-4 categories revealed no significant differences across mild (39.1 ± 29.9 pg/mL), moderate (36.9 ± 27.04 pg/mL), and severe (45.4 ± 36.2 pg/mL) fibrosis (*p* = 0.83). ASCVD risk categories demonstrated no significant TNF-α variation across low (37.9 ± 28.5 pg/mL), borderline (34.5 ± 34.7 pg/mL), intermediate (44.9 ± 29.4 pg/mL), and high (59.5 ± 31.6 pg/mL) risk groups (*p* = 0.46) [Table S3].

### 3.7. Comprehensive Summary of Inflammatory Markers and Fibrosis Severity

The collective findings from Section 3.4, Section 3.5 and Section 3.6 establish a consistent pattern: none of the three inflammatory markers (IL-6, hsCRP, TNF-α) demonstrated significant associations with liver disease severity across any of the five non-invasive fibrosis scoring systems (CAP, APRI, NAFLD-F, TE, FAST) or with cardiovascular risk assessment (ASCVD score). This consistent lack of correlation across multiple validated assessment tools strengthens the evidence that systemic inflammation is decoupled from hepatic fibrosis progression in MASLD

### 3.8. Correlations Between Inflammatory Markers and Metabolic Parameters

In contrast to the absence of associations with fibrosis severity, inflammatory markers demonstrated significant correlations with metabolic parameters, suggesting that their primary role reflects metabolic dysfunction rather than liver disease progression [Table S4].

**Figure 2:**
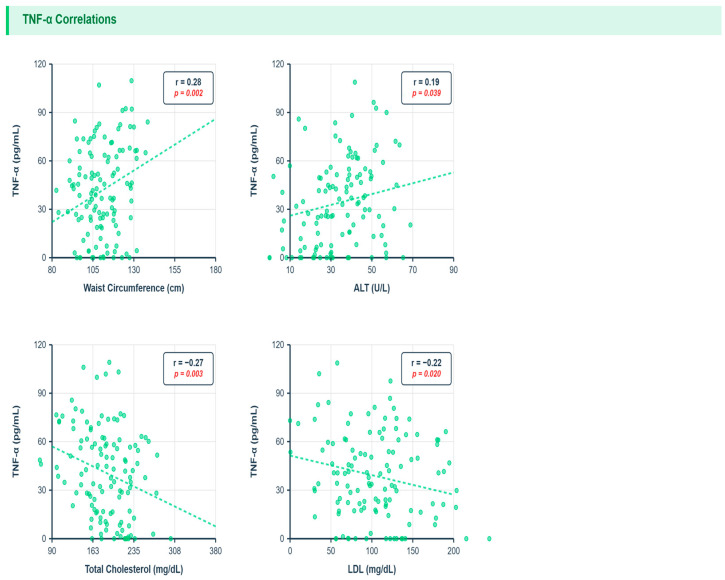
Correlations Between TNF alpha and Metabolic Parameters.

TNF-α showed significant positive correlations with waist circumference (r = 0.28, *p* = 0.002) and ALT (r = 0.019, *p* = 0.03), indicating associations with central obesity and hepatocellular injury. Notably, TNF-α demonstrated significant inverse correlations with total cholesterol (r = −0.27, *p* = 0.003) and LDL-cholesterol (r = −0.22, *p* = 0.02), as well as with triglycerides (r = −0.13, *p* = 0.015). These divergent lipid associations suggest complex interactions between TNF-α-mediated inflammation and lipid metabolism [Figure 2].

**Figure 3:**
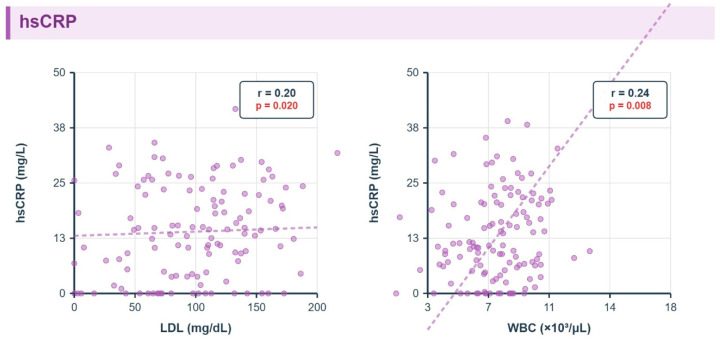
Correlations Between Hs-CRP and Metabolic Parameters.

HsCRP demonstrated a distinct pattern of metabolic associations, showing significant positive correlations with LDL-cholesterol (r = 0.20, *p* = 0.02) and white blood cell count (r = 0.24, *p* = 0.008), reflecting its role as a marker of cardiovascular risk and systemic inflammation [Figure 3].

IL-6 showed no significant correlations with any of the measured anthropometric or metabolic parameters, including age, waist circumference, BMI, fasting blood glucose, HOMA-IR, liver enzymes, lipid profile components, or hematological parameters (all *p* > 0.05). This lack of correlation may reflect measurement variability or distinct biological roles compared to TNF-α and hsCRP in this population.

No significant correlations were observed between any inflammatory markers and age, BMI, fasting blood glucose, HOMA-IR, AST, GGT, albumin, HDL-cholesterol, hemoglobin, or platelet count (all *p* > 0.05) [Table S4]. These findings collectively indicate that systemic inflammatory markers in MASLD showed stronger associations with metabolic disturbances, particularly central obesity and lipid dysregulation, rather than hepatic fibrosis severity.

Figure 2: TNF-α vs. Metabolic Parameters: X-axis: waist circumference (cm), total cholesterol (mg/dL); Y-axis: TNF-α (pg/mL). Points colored by TE fibrosis (blue = mild, orange = moderate, red = severe). No fibrosis severity gradient (overlap across colors); positive waist trend (r = 0.28, q = 0.01), inverse cholesterol (r = −0.27, q = 0.03).

Figure 3: hsCRP vs. Metabolic Parameters: Similar format. hsCRP rises with LDL (r = 0.20, q = 0.02); no fibrosis pattern.

### 3.9. Metabolic and Multivariate Associations

Univariate Spearman’s correlations (post-FDR q < 0.05):

- TNF-α: waist circumference r = 0.28 q = 0.01, ALT r = 0.19 q = 0.04; inverse total cholesterol r = −0.27 q = 0.03, LDL r = −0.22 q = 0.04, triglycerides r = −0.13 q = 0.045 (corrected).
- hsCRP: LDL r = 0.20 q = 0.02, WBC r = 0.24 q = 0.01.
- IL-6: no significant metabolic correlations post-FDR. Table 3 and Table 4

## 4. Discussion

MASLD is now recognized as the most prevalent chronic liver disease worldwide, representing a multisystem metabolic disorder in which inflammatory cascades play crucial roles in both pathogenesis and prognosis [24,25]. We conducted a cross-sectional observational study of 120 patients to evaluate inflammatory markers as potential prognostic indicators. Our principal finding reveals that systemic inflammatory markers correlate more strongly with metabolic parameters than with non-invasive indicators of fibrosis severity, suggesting a fundamental shift from fibrosis staging to metabolic-inflammatory interactions in MASLD. This unexpected dissociation between inflammation and fibrosis severity forms the foundation for reconsidering how we interpret inflammatory biomarkers in clinical practice.

This cross-sectional analysis demonstrates that systemic inflammatory markers (IL-6, TNF-α, hsCRP) in MASLD patients showed no significant associations with fibrosis severity across five validated non-invasive scoring systems, but exhibited consistent correlations with metabolic parameters that persisted after multivariate adjustment. These findings highlight a dissociation between circulating inflammation and hepatic fibrosis, with stronger links to metabolic dysfunction (e.g., TNF-α with waist circumference β = 0.28, *p* < 0.01; hsCRP with LDL β = 0.18, *p* = 0.03).

These findings differ from those of studies that identified elevated inflammatory markers associated with advanced fibrosis stages [26,27,28], which naturally raises the question: why this divergence? Several methodological factors offer plausible explanations. The lack of fibrosis associations aligns with the cohort’s composition: 66.7% had mild fibrosis by transient elastography, with only 10% severe and 2–5% advanced disease across other scores. This skewed distribution substantially limited statistical power to detect inflammation-fibrosis links in advanced stages, restricting conclusions primarily to early MASLD. FAST score analyses (99.2% mild, n = 1 severe) were descriptive only due to extreme imbalance.

Studies reporting positive correlations often included higher proportions of advanced fibrosis, creating greater phenotypic variability that facilitates detection of biomarker–severity relationships [29]. Additionally, we utilized non-invasive assessment methods rather than liver biopsy. While extensively validated and recommended by current guidelines [30], these tools may capture different aspects of liver pathology compared to histological assessment. Furthermore, unmeasured confounding variables such as dietary patterns, physical activity, ethnic background, and genetic factors which are known to influence both steatosis development and inflammatory responses [31] may have contributed to observed patterns. Recent cluster analyses have revealed substantial heterogeneity within MASLD, identifying distinct molecular subtypes with different inflammatory signatures and progression risks [29,32], suggesting that our cohort may represent a specific phenotype where metabolic rather than hepatic factors dominate the inflammatory profile.

Despite these methodological considerations, our findings align with a growing body of evidence suggesting that systemic inflammatory markers in MASLD may be more closely linked to metabolic dysfunction than histological progression. While hsCRP has been consistently identified as a major risk factor for NAFLD development [33,34], its association with fibrosis severity shows considerable variability [35]. Similarly, findings regarding TNF-α remain inconsistent across prospective and cross-sectional studies [36,37,38], likely reflecting the complex, multifaceted role of inflammatory mediators that function simultaneously as drivers of metabolic dysfunction, hepatic injury, and systemic complications. This complexity led us to investigate where, precisely, the inflammatory signals might be originating [5,39,40,41].

The adipose–liver axis emerged as a compelling explanation for our observations. The strong positive association between TNF-α and waist circumference observed in our study directly supports the adipose-inflammation connection, consistent with meta-analyses demonstrating robust correlations between TNF-α and central obesity [42,43,44]. Central adiposity is characterized by increased macrophage infiltration, adipocyte hypertrophy, and altered adipokine secretion that collectively drive systemic inflammation [7]. This finding carries a clear clinical implication: addressing central obesity through lifestyle modifications, pharmacotherapy, or bariatric interventions may be more effective at reducing systemic inflammation than liver-targeted therapies alone. Building on this adipose-centric perspective, we also observed that the association between TNF-α and ALT likely results from TNF-α’s direct hepatotoxic effects and disruption of insulin signaling pathways [45,46]. However, and this is crucial, this association with liver injury markers did not translate into associations with fibrosis severity, suggesting that while TNF-α may contribute to hepatocellular damage, its elevation primarily reflects metabolic dysfunction rather than fibrogenic activity [47,48]. This distinction became even more intriguing when we examined the inflammatory markers’ relationships with lipid profiles.

Another unexpected layer of complexity is that we observed an inverse association between TNF-α and both total cholesterol and LDL-cholesterol, as well as with triglycerides, a pattern that initially appears counterintuitive given the traditional association between inflammation and dyslipidemia in metabolic syndrome. Yet this paradoxical relationship can be explained through well-established mechanisms of TNF-α-mediated effects on cholesterol homeostasis: TNF-α enhances LDL receptor expression on endothelial cells and macrophages, accelerating LDL internalization [49], downregulates HMG-CoA reductase [50], and facilitates PCSK9-mediated degradation of LDL receptors [51,52]. Clinical evidence from anti-TNF-α medications confirms that active TNF-α signaling suppresses circulating LDL-cholesterol [53], carrying important therapeutic implications: anti-inflammatory strategies targeting TNF-α pathways may inadvertently worsen lipid profiles, necessitating concurrent lipid management.

In striking contrast, hsCRP showed the opposite pattern, correlating positively with LDL-cholesterol and white blood cell count, likely reflecting chronic low-grade inflammation combined with the lipid dysfunction characteristic of metabolic syndrome [54,55,56]. The landmark 30-year study by Ridker and colleagues demonstrated that hsCRP was a stronger predictor of cardiovascular events than LDL-cholesterol, with highest risk in individuals with elevated levels of both biomarkers [57]. This is particularly relevant to MASLD, where cardiovascular disease represents the primary cause of mortality, and patients with elevated hsCRP and LDL simultaneously face substantially higher cardiovascular event rates [58,59,60,61]. These divergent associations suggest that TNF-α and hsCRP represent distinct inflammatory mechanisms requiring different therapeutic approaches [62,63], supporting a precision medicine approach where strategies are tailored to individual inflammatory-metabolic signatures. This mechanistic heterogeneity has profound implications for how we should interpret and act upon elevated inflammatory markers in clinical practice.

These observations compel us to reconsider the clinical utility of inflammatory markers in MASLD management. When elevated inflammatory markers are detected, clinicians should interpret these primarily as indicators of metabolic dysfunction and cardiovascular risk rather than advanced liver disease. This interpretation aligns with the emerging conceptualization of MASLD as a systemic metabolic disorder with cardiovascular and malignant complications extending beyond hepatic pathology [64,65]. Given that cardiovascular disease accounts for the majority of deaths in MASLD patients [24], elevated inflammatory markers particularly hsCRP should prompt comprehensive cardiovascular risk assessment using validated tools such as the ASCVD calculator and guide intensification of preventive strategies [66]. Our findings therefore support what might be termed a dual-pathway approach to MASLD management, addressing both metabolic and hepatic components independently [67]. Our data suggest the need for parallel assessment of metabolic-inflammatory status alongside hepatic fibrosis evaluation.

When we consider the therapeutic implications of our findings, recent landmark trials offer encouraging validation of this metabolic-focused approach. These trials have fundamentally shifted MASLD treatment toward targeting metabolic dysfunction, exemplified by semaglutide’s success in achieving significant NASH resolution through weight loss and improved insulin sensitivity. The field continues to advance with combination strategies, such as pairing GLP-1 agonists with FGF21 analogues or SGLT2 inhibitors, to synergistically address systemic metabolism and direct anti-inflammatory effects [68,69,70]. This therapeutic evolution reinforces that effective treatment must tackle the root metabolic drivers of inflammation, though patients with advanced fibrosis likely need combined metabolic and anti-fibrotic approaches to achieve comprehensive disease modification.

Several limitations temper the interpretation of our results. The cross-sectional design of this study precludes causal or longitudinal inferences. Univariate correlations were supplemented with multivariate models adjusting for age, sex, BMI, waist, diabetes, and liver enzymes, but complete adjustment for medications (statins 23.3%, diabetes drugs 26.7%) was limited by collinearity; smoking data (15%) recorded but not fully modeled. Marker elevations were relative to published reference ranges, not a matched control group from the same population. Absence of liver histology represents a key gap, as non-invasive scores have imperfect correlation with fibrosis stage.

These results suggest that inflammatory markers have limited utility as surrogates for fibrosis severity in early MASLD, instead signaling underlying metabolic and cardiometabolic risk. Clinically, this supports decoupling inflammation management from fibrosis assessment: target metabolic drivers (obesity, dyslipidemia) independently while using dedicated fibrosis tools (FIB-4, TE) for progression monitoring. Future prospective studies in advanced fibrosis cohorts, with matched controls and serial measurements, are needed to confirm if this dissociation holds across disease stages.

## 5. Conclusions

In this cross-sectional MASLD cohort predominantly comprising early-stage disease, systemic inflammatory markers showed no associations with non-invasive fibrosis scores but correlated independently with metabolic parameters after adjustment. These findings indicate that markers are primarily associated with metabolic dysfunction rather than hepatic fibrosis severity, indicating a need for separate targeting of metabolic and fibrotic pathways in MASLD management.

## Figures and Tables

**Table 1:** Demographic characteristics and laboratory investigations among study group.

Variables	Number (n = 120)	
	Mean ± SD	Median (Range)
Age (years)	47.3 ± 7.9	47.5(29–66)
Sex	No.	%
Male	52	43.3%
Female	68	56.7%
Diabetes mellitus		
No	84	70%
Yes	36	30%
Anthropometric measure	Mean ± SD	Median (Range)
Waist circumference (cm)	111.4 ± 11.9	110 (84–176)
BMI (kg/m^2^)	35.2 ± 7.3	34 (24.4–61.8)
Laboratory investigations	Mean ± SD	Median (Range)
FBS	120.02 ± 47.9	105 (60–372)
HOMA-IR	2.2 ± 0.98	1.9 (0.86–6.4)
ALT	33.1 ± 14.1	28 (16–85)
AST	33.5 ± 11.2	31 (15–75)
GGT	48.9 ± 38.9	38 (14–251)
Albumin	4.02 ± 0.33	4 (3–4.7)
Total cholesterol	191.1 ± 46.3	191 (99–376)
Triglyceride	196.3 ± 101.2	172.5 (66–797)
LDL	103.6 ± 47.7	101 (3.2–378)
HDL	47.6 ± 9.9	47.5 (26–85)
Hemoglobin	13.3 ± 1.5	13.3 (10–16.5)
PLT count	271.4 ± 59.5	266.5 (151–422)
WBCs	6.9 ± 2.3	6.1 (3.7–16.7)

**Table 2:** Assessment of grades of MASLD severity according to the measured scores among study groups.

Variables	MASLD Severity (n = 120)					
	Mean ± SD				Median (Range)	
CAP score	325.7 ± 54.2				334 (100–400)	
TE score	7.5 ± 4.3				6.1 (2.1–28)	
FAST score	0.50 ± 1.6				0.33 (0.04–18)	
APRI score	0.33 ± 0.15				0.30 (0.1–1.1)	
Fib 4 score	1.09 ± 0.54				1.01 (0.43–3.96)	
NAFLD F score	−1.17 ± 1.24				−1.17 (−3.86/2.15)	
ASCV score	3.8 ± 5.02				2 (0.3–35.3)	
Severity grades	Mild		Moderate			Severe
TE score	80 (66.7%)		28 (23.3%)			12 (10%)
FAST score	119 (99.2%)		1 (0.8%)			----
Fib 4 score	86 (71.7%)		31 (25.8%)			3 (2.5%)
	Low		Intermediate			High
APRI score	114 (95%)		6 (5%)			----
NAFLD F score	46 (38.3%)		39 (32.5%)			35 (29.2%)
	S0	S1		S2		S3
CAP score	8 (6.7%)	4 (3.3%)		8 (6.7%)		100 (83.3%)
	Low	Borderline		Intermediate		High
ASCV score	96 (80%)	11 (9.2%)		10 (8.3%)		3 (2.5%)

**Table 3:** Key Correlations (FDR-adjusted).

Marker	Parameter	r	*p*	q (FDR)
TNF-α	Waist (cm)	0.28	0.002	0.01
TNF-α	Triglycerides (mg/dL)	−0.13	0.015	0.045
hsCRP	LDL (mg/dL)	0.20	0.02	0.02
hsCRP	WBC (10^9^/L)	0.24	0.008	0.01

Fibrosis scores: all q > 0.20 (NS).

**Table 4:** Multivariate Linear Regression Models.

Model	Dependent	Predictor	β	*p*	Adjusted R^2^
1	TNF-α (pg/mL)	Waist (cm)	0.28	<0.01	0.32
		Diabetes (yes)	0.15	0.08	
		TE (kPa)	0.07	0.32	
2	hsCRP (mg/L)	LDL (mg/dL)	0.18	0.03	0.25
		BMI (kg/m^2^)	0.12	0.15	
		FIB-4	0.05	0.48	

Fibrosis scores nonsignificant across models (all *p* > 0.20).

## Data Availability

Raw data supporting this study’s findings are available from the corresponding author upon reasonable request.

## Supplementary Materials

The following supporting information can be downloaded at: https://www.mdpi.com/article/10.3390/metabo16010025/s1, Table S1: Comparisons of IL6 level in MASLD severity degrees by different scores among study groups; Table S2:Comparisons of Hs-CRP level in MASLD severity degrees by different scores among study groups.; Table S3: Comparisons of TNF-a level in MASLD severity degrees by different scores among study groups; Table S4: Correlation between Inflammatory markers with laboratory data among cases.
