# Supplementary material for: Associations Between Systemic Inflammatory Markers, Metabolic Dysfunction, and Liver Fibrosis Scores in Patients with MASLD
Source: Metabolites. 2025 Dec 25;16(1):25. doi: 10.3390/metabo16010025 (PMC12843941; doi:10.3390/metabo16010025)
Supplement: Supplementary file 1 [file metabolites-16-00025-s001.zip › metabolites-4041609-supplementary.pdf]

Table S1: Comparisons of IL6 level in MASLD severity degrees by different scores among study groups

Variables	Inflammatory markers IL6		
	Mean ± SD	Median (Range)	P-value
**CAP score**			
S0	13.6±3.2	13.6(9.1-17.5)	0.86
S1	16.3±11.3	16.5(3.7-28.5)	
S2	12.5±7.1	14.4(0-20.9)	
S3	15.4±9.8	14.5(0-68.3)	
**APRI score**			
Low	14.9±9.5	14.4(0-68.3)	0.22
Intermediate	17.1±3.5	16.2(13.6-21.2)	
**NAFLD F score**			
Low	15.9±11.7	13.7(0-68.3)	0.70
Intermediate	13.6±6.9	14.4(0-26.4)	
High	15.5±8.2	15.1(0-34.1)	
**TE score**			
Mild	14.8±10.3	14.2(0-68.3)	0.75
Moderate	14.7±7.4	13.5(0-34.1)	
Severe	17.8±6.4	15.2(10.3-31.1)	
**FAST score**			
Mild	15.1±9.4	14.4(0-68.3)	0.73
Severe	12.7±0.0	12.7(12.7-12.7)	
**Fib 4 score**			
Mild	14.8±9.8	13.4(0-68.3)	0.19
Moderate	15.4±8.2	15.1(0-34.1)	
Severe	19.3±4.4	20.9(14.3-22.6)	
**ASCV score**			
Low	14.3±8.7	13.8(0-68.3)	0.15
Border line	15.8±14.4	15.7(0-47.6)	
Intermediate	18.7±7.2	16.8(9.8-28.5)	
High	22.9±9.8	18.9(15.9-34.1)	

Table S2: Comparisons of Hs-CRP level in MASLD severity degrees by different scores among study groups.

Variables	Inflammatory markers Hs-CRP		
	Mean ± SD	Median (Range)	P-value
**CAP score**			

S0	19.1±19.9	9.5(0.5-44.9)	0.69
S1	6.7±3.8	6.8(3-10)	
S2	15.2±13.4	14.7(1.6-43.4)	
S3	11.8±10	8(1-43.5)	
**APRI score**			
Low	12.4±11.3	7.6(0.5-44.9)	0.42
Intermediate	11.8±4.7	12.6(4.2-18)	
**NAFLD F score**			
Low	15.7±13.9	9.8(0.5-44.9)	0.16
Intermediate	9.6±7.9	7.5(1.5-30)	
High	10.9±8.5	10.5(1-40.2)	
**TE score**			
Mild	12.6±11.3	7.6(0.5-43.4)	0.73
Moderate	12.1±10.2	11.5(1-43.5)	
Severe	10.9±12.1	6.8(1.5-44.9)	
**FAST score**			
Mild	12.4±11.1	8.4(0.5-44.9)	0.48
Severe	3.9±0.0	3.9(3.9-3.9)	
**Fib 4 score**			
Mild	13.1±11.7	8(0.5-44.9)	0.38
Moderate	10.1±9.4	8.4(1.5-43.5)	
Severe	12±5.9	14.2(5.3-16.5)	
**ASCV score**			
Low	12.8±11.7	8(0.5-44.9)	0.77
Border line	13.5±10.3	12.8(2.8-30)	
Intermediate	8.3±4.4	8.8(2.4-14.2)	
High	7.8±6.9	5.3(2.5-15.7)	

Table S3: Comparisons of TNF-a level in MASLD severity degrees by different scores among study groups.

Variables	Inflammatory markers		
	TNF-a		
	Mean ± SD	Median(Range)	P-value
**CAP score**			
S0	42.02±35.1	30.9(0-90.5)	0.91
S1	34.1±20.3	38.2(6.5-43.4)	
S2	32.2±24.7	22.5(4.8-69.3)	
S3	39.2±29.5	30.3(0-109.6)	
**APRI score**			
Low	38.8±29.3	30.4(0-109.6)	0.91
Intermediate	36.8±27.8	25.7(15.3-87.2)	
**NAFLD F score**			
Low	42.1±31.01	30.6(0-109.6)	0.45
Intermediate	33.6±27.4	29.7(0-103)	

High	39.9±28.3	31.9(0-90.5)	
**TE score**			
Mild	36.1±30.2	24.9(0-109.6)	0.17
Moderate	43.1±26.9	39.7(2.9-94.8)	
Severe	45.8±26.4	43.9(0-87.2)	
**FAST score**			
Mild	38.3±28.9	30.4(0-109.6)	0.17
Severe	86.6±0.0	86.6(86.6-86.6)	
**Fib 4 score**			
Mild	39.1±29.9	30.6(0-109.6)	0.83
Moderate	36.9±27.04	24.4(0-91.9)	
Severe	36.2±26.8	22.3(87.2-)	
**ASCV score**			
Low	37.9±28.5	29.9(0-103)	0.46
Border line	34.5±34.7	26.8(0-109.6)	
Intermediate	44.9±29.4	44.3(0-87.2)	
High	59.5±31.6	68(24.4-86)	

Table S4: Correlation between Inflammatory markers with laboratory data among cases.

Variables (n=120)	Inflammatory markers					
	IL6		TNF-a		Hs-CRP	
	r	P-value	r	P-value	r	P-value
Age (years)	0.09	0.28	0.00	0.99	-0.03	0.69
WC(cm)	0.14	0.12	**0.28**	**0.002***	-0.01	0.91
BMI (kg/m2)	0.03	0.73	-0.05	0.59	0.16	0.08
**Laboratory investigations**						
FBS	-0.08	0.4	0.06	0.54	-0.06	0.55
HOMA –IR	-0.06	0.48	0.04	0.69	0.06	0.52
ALT	0.03	0.77	**0.019**	**0.03***	0.02	0.80
AST	0.08	0.36	0.02	0.82	0.12	0.21
GGT	-0.06	0.48	0.004	0.96	0.06	0.51
Albumin	-0.01	0.89	0.04	0.63	-0.01	0.88
Total cholesterol	0.01	0.89	**-0.27**	**0.003***	0.09	0.29
Triglyceride	0.09	0.35	-0.13	0.015	-0.17	0.07
LDL	-0.02	0.81	**-0.22**	**0.02***	**0.20**	**0.02***
HDL	-0.007	0.93	-0.09	0.36	0.06	0.51
Hemoglobin	-0.04	0.67	-0.05	0.60	-0.003	0.97
PLT count	-0.13	0.16	0.04	0.63	0.016	0.08
WBCs	0.06	0.50	-0.06	0.50	**0.24**	**0.008***
